# COVID19-associated new-onset movement disorders: a follow-up study

**DOI:** 10.1007/s00415-023-11661-x

**Published:** 2023-03-21

**Authors:** Susanne A. Schneider, Soaham Desai, Onanong Phokaewvarangkul, Elena Cecilia Rosca, Jirada Sringean, Pria Anand, Gary Álvarez Bravo, Francisco Cardoso, Anna M. Cervantes-Arslanian, Harshad Chovatiya, David Crosiers, Femke Dijkstra, Conor Fearon, Francisco Grandas, Eric Guedj, Antonio Méndez-Guerrero, Muhammad Hassan, Joseph Jankovic, Anthony E. Lang, Karim Makhoul, Lorenzo Muccioli, Sarah A. O’Shea, Vahid Reza Ostovan, Javier Ricardo Perez-Sanchez, Ritesh Ramdhani, Victoria Ros-Castelló, Christina Schulte, Priyank Shah, Lars Wojtecki, Pramod Kumar Pal

**Affiliations:** 1 Department of Neurology, Ludwig Maximilians University Munich, Marchioninistr. 15, 81377 Munich, Germany; 2 Department of Neurology, Shree Krishna Hospital, Pramukhswami Medical College, Bhaikaka University, Karamsad, Anand, Gujarat India; 3 Chulalongkorn Centre of Excellence for Parkinson’s Disease and Related Disorders, Faculty of Medicine, Chulalongkorn University and King Chulalongkorn Memorial Hospital, 1873 Rama 4 Road, Bangkok, Thailand; 4 Department of Neurology, Victor Babes University of Medicine and Pharmacy, Timisoara, Romania; 5 Departments of Neurology, Neurosurgery, and Medicine Boston University School of Medicine and Boston Medical Center, Boston, USA; 6 Neuroimmunology and Multiple Sclerosis Unit of Girona, University Hospital Dr. Josep Trueta of Girona, Girona, Catalonia Spain; 7 Movement Disorders Unit, Neurology Service, Internal Medicine Department, UFMG, Belo Horizonte, Brazil; 8 Faculty of Medicine and Health Sciences, Translational Neurosciences, University of Antwerp, Antwerp, Belgium; 9 Department of Neurology, Antwerp University Hospital, Antwerp, Belgium; 10 Division of Neurology, Edmond J. Safra Program in Parkinson’s Disease, Morton and Gloria Shulman Movement Disorders Clinic, Toronto Western Hospital – UHN, University of Toronto, Toronto, ON Canada; 11 Movement Disorders Unit, Neurology Department, Hospital General Universitario Gregorio Marañón, Madrid, Spain; 12 Institut Fresnel, Nuclear Medicine Department, Aix Marseille Univ, APHM, CNRS, Centrale Marseille, Timone Hospital, CERIMED, Marseille, France; 13 Neurology Department, Hospital 12 de Octubre, Madrid, Spain; 14 Shaheed Zulfiqar Ali Bhutto Medical University, Islamabad, Pakistan; 15 Parkinson’s Disease Center and Movement Disorders Clinic, Department of Neurology, Baylor College of Medicine, Houston, TX USA; 16 Department of Biomedical and Neuromotor Sciences, University of Bologna, Bologna, Italy; 17 Department of Neurology, Columbia University Vagelos College of Physicians and Surgeons, New York, USA; 18 Clinical Neurology Research Center, Shiraz University of Medical Sciences, Shiraz, Iran; 19 Zucker School of Medicine at Hofstra/Northwell, Hempstead, NY USA; 20 Neurology Department, Ramon y Cajal University Hospital, Madrid, Spain; 21 Departmemt of Neurology and Neurorehabilitation, Hospital Zum Heiligen Geist, Academic Teaching Hospital of the Heinrich-Heine-University Duesseldorf, Kempen, Germany; 22 Privya Neurology Clinic, Ahmedabad, Gujarat India; 23 Institute of Clinical Neuroscience and Medical Psychology, Medical Faculty, Heinrich-Heine-University Düsseldorf, Düsseldorf, Germany; 24 Department of Neurology, National Institute of Mental Health and Neurosciences (NIMHANS), Bangalore, Karnataka India

**Keywords:** COVID, Sars-CoV2, Movement disorder, Long covid, Outcome, Myoclonus ataxia, Parkinsonism

## Abstract

**Background:**

Neurological symptoms are common manifestation in acute COVID-19. This includes hyper- and hypokinetic movement disorders. Data on their outcome, however, is limited.

**Methods:**

Cases with new-onset COVID-19-associated movement disorders were identified by searching the literature. Authors were contacted for outcome data which were reviewed and analyzed.

**Results:**

Movement disorders began 12.6 days on average after the initial onset of COVID-19. 92% of patients required hospital admission (mean duration 23 days). In a fraction of patients (6 of 27; 22%; 4 males/2 females, mean age 66.8 years) the movement disorder (ataxia, myoclonus, tremor, parkinsonism) was still present after a follow-up period of 7.5 ± 3 weeks. Severe COVID-19 in general and development of encephalopathy were risk factors, albeit not strong predictors, for the persistence.

**Conclusions:**

The prognosis of new-onset COVID-19-associated movement disorder appears to be generally good. The majority recovered without residual symptoms within several weeks or months. Permanent cases may be due to unmasking of a previous subclinical movement disorder or due to vascular/demyelinating damage. Given the relatively low response rate of one third only and the heterogeneity of mechanisms firm conclusions on the (long-term) outome cannot, however, be drawn.

**Supplementary Information:**

The online version contains supplementary material available at 10.1007/s00415-023-11661-x.

## Introduction

Severe acute respiratory syndrome coronavirus 2 (SARS-CoV2, 2019-nCoV) emerged in December 2019 and spread into a worldwide pandemic. More than 641 million people worldwide have been verifiably infected with Sars-CoV-2 (as of November 2022). About 10–30% of COVID-19 patients develop neurological symptoms in the acute phase, including a broad range of new-onset movement disorders [1]. Among these, ataxia and myoclonus are the most common manifestation; however, parkinsonism, dystonia, chorea and other movement disorders may also occur [2]. Little is known about the outcome of these patients, as most case reports were published relatively soon after the occurrence of symptoms. Long-term data are lacking. As a mission of the International Parkinson and Movement Disorders Society (MDS) Infection-Related Movement Disorder (IRMD) Study Group, we therefore collected detailed clinical information and outcome data of COVID-19-associated movement disorder cases.

## Methods

We searched the literature using PubMed and Medrxiv, the preprint server for health sciences, to identify cases of COVID-19-associated new-onset movement disorders published until February 2022. We used the (combination of) search terms: movement disorder; parkinsonism/Parkinson’s disease; dystonia; chorea; tics; myoclonus; SARS-CoV2, and COVID-19. Further articles were identified by cross-referencing. The authors were contacted and asked to provide follow-up information using a standardized questionnaire. Additional cases were also accepted, including by submission of data via a publicly available page on the website of the International Parkinson Disease and Movement Disorders Society (https://www.movementdisorders.org/COVID-19-Pandemic-MDS/COVID-19-Repository-Submissions.htm).

## Results

**Fig. 1:**
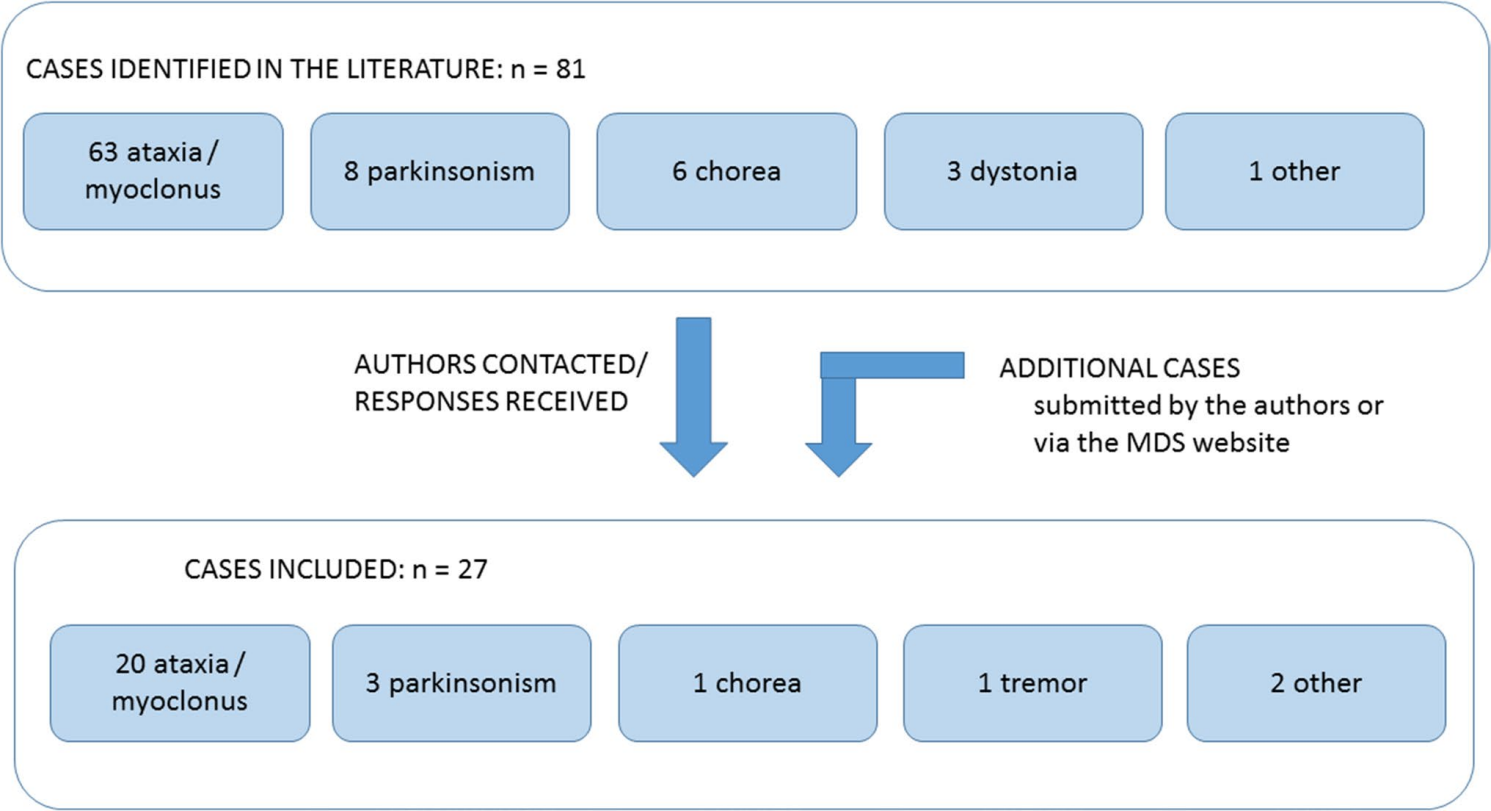
Identification of cases

Reports of 81 patients with new-onset movement disorders were identified (63 ataxia/myoclonus, 8 parkinsonism, 6 chorea, 3 dystonia, other movement disorders), the authors of which were contacted by email. Information was received on 27 patients (8 female, 19 male) who were included in the final analysis (20 with ataxia/myoclonus [3–12], 3 with a parkinsonian syndrome [13–15], 1 with chorea [16], 1 with tremor, 1 with limb tremor and gait ataxia [17]), yielding an overall response rate of 33.3%. (Fig. 1) Submissions were received from the US, Spain, India (5 cases each), Germany (4 cases), Canada (2 cases), Pakistan (2 cases) and Italy, Belgium, France and Iran (1 case each). None of the cases had been vaccinated against COVID-19.

The clinical information is summarized in Table 1. Briefly, the mean age at movement disorder onset for the whole group was 56.8 ± 12.6 years.

**Table 1:** Clinical description of the study cohort (*n* = 27)

Movement Disorder	Number of patients	Age	Sex (M/F)	Days of Hospital Stay	Days Between COVID and movement disorder	Follow Up^#^ [weeks]
Myoclonus	7	58.7 ± 13.1	3/4	20.4 ± 12.1	0,2,4,7,9,25,35	56.7 ± 32.3
Myoclonus-Ataxia	5	57.2 ± 12.4	4/1	12.2 ± 8.1	10,12,14,17; unknown in one case	52.4 ± 31.2
Ataxia	5	53.2 ± 11.4	3/2	16.2 ± 10.2	0, 3, 12, 21; unknown in one case	67.6 ± 20.1
Opsoclonus-Myoclonus-Ataxia	3	46.3 ± 13.0	3/0	5.3 ± 0.5	8, 10, 21	54.7 ± 3,8
Hypokinetic rigid syndrome	3	56 ± 7.5	2/1	48.3 ± 40.0	5, 33; ?44*	67.0 ± 21.2
Tremor	1	62	1/0	37	10	8
Serotonin Syndrome	1	66	1/0	33	3	54
Limb tremor and gait ataxia	1	78	1/0	90	10	81
Chorea	1	58	1/0	13	2	3

^*^Onset not clear as the patient was intubated; movement disorder was first observed when the patient was extubated on day 44; # standardised from the outset

Severity of the COVID-19 infection ranged from mild to severe requiring hospital admission in all but two cases (92.6%; exceptions: one case with ataxia myoclonus and one with parkinsonism) with a mean cycle threshold score of 8.6 (data available for 18 patients) (with high scores indicating a low concentration of viral genetic material which is typically associated with a lower risk of infectivity).

The movement disorder developed an average of 12.6 ± 9.21 days after the initial onset of COVID-19 (information available for 24 patients). It was still persistent in six cases (four males, two females, mean age 66.8 years) after a mean duration of follow-up of 53 ± 23 days. Clinical details of these six cases are presented in Table 2. Briefly, their movement disorders manifested as ataxia, myoclonus, myoclonus-ataxia, tremor and parkinsonism. The movement disorder resolved in the remaining 21 patients within several weeks.

**Table 2:** Summary of cases with persistent movement disorders

	Makhoul and Jankovic [14]	Makhoul and Jankovic (unpublished)	Muccioli et al. [7]	Desai and Chovatiya (unpublished)	Anand et al. [11], case 8	Fearon et al. [15]
Diagnosis	Unmasking of underlying still non-symptomatic iPD	Tremor	Immune‐mediated myoclonus	Post-stroke cerebellar ataxia	Myoclonic encephalopathy	Hypoxic–ischemic parkinsonism
Phenotype	Parkinsonism	Tremor	Myoclonus	Ataxia	Ataxia myoclonus, tremor, dystonia	Parkinsonism
Age	64	62	61	67	71	46
Sex	F	M	M	F	M	M
Admission required	No	Yes	Yes	Yes	Yes	Yes
Days in hospital	0	37	38	17	18	98
ICU care	No	Yes	Yes	Yes	No	Yes
BPAP	No	Yes	Yes	Yes	No	No
Previously vaccinated	No	No	No	No	No	No
Days between infection and onset of mov disord	5	10	25	21	7	44*
Imaging findings	Decreased putaminal uptake on DaTscan	Brain MRI normal	Moderate cerebral small vessel disease	Cerebellar infarct	Diffuse frontal and temporal pachymeningeal enhancement, with resolution on follow-up imaging	Edema and haemorrhage, globus pallidus and dentates bilaterally, diffuse white matter changes
Treated with immune-modulatory drugs	No	Yes, steroids	No	Yes, steroids	No	No
Symptomatic treatment	Carbidopa/ levodopa, rasagiline	Propranolol, primidone, levetiracetam	Levetiracetam, clonazepam	None	Levetiracetam, valproic acid, zonisamide	Carbidopa/ levodopa
Course of mov. disord.	Steadily better	Steadily better	Steadily better	Steadily better	Steadily better	Static
Available follow-up (months)	30	2	17	54	12	12
Video previously published	Yes	No, published here (Video1)	Yes	No	No, published here (Video2)	Yes

^*^Notably, this patient was intubated from day 5 after symptom onset until day 44

The mean duration of hospital admission was 22.9 ± 23.8 days for all cases (19.6 days for those who recovered vs 34.7 days for those with a persistent movement disorder). Oxygen supply was required in 51.8% of cases (56% of those admitted). Patients with an akinetic rigid syndrome stayed longer (48.3 ± 40 days) than patients with other phenotypes. Eight patients needed ICU treatment (29.6% of all cases; 32% of those admitted; 51.14% of those who needed oxygen; 19% of those who recovered vs 66.7% of those with a persistent movement disorder). Five patients required biphasic positive airway pressure (BPAP)(20% of those admitted); six patients were intubated (24% of those admitted).

Fourteen patients were treated with immunomodulatory drugs including steroids (nine patients), intravenous immunoglobulin (three patients) and a combination (one case). Most of the patients received symptomatic treatment for the particular type of movement disorder for a short period. At last follow-up, only two patients with parkinsonism continued to receive levodopa/carbidopa and only three patients with myoclonus were continuing to take levetiracetam and clonazepam. All other patients in our study did not require any symptomatic medicines for their movement disorders. One case [17] developed serotonin syndrome triggered by lopinavir/ritonavir (LPV/r) antiretroviral treatment.

Common comorbidities were present more frequently in those with a persistent movement disorder, i.e. hypertension (present in 83% of cases vs 43% of those who recovered); cardiac disease (14% vs 33%); cancer (33% vs 4%).

Brain imaging was performed in all cases and was reported to be normal in 63%. Abnormalities were detected in five cases whose movement disorder subsided, i.e. hemorrhagic leukoencephalitis (ataxia), bilateral cerebellar hyperintensities with cortical-meningeal enhancement (ataxia), cortical and brainstem ischemic lesions (myoclonus) and periventricular ischemic changes (chorea/myoclonus). On follow-up, the hemorrhagic changes had evolved into cerebellar gliosis and subcortical leukariosis, whereas the cerebellar edema and enhancement in the other ataxia patient completely resolved. No change was seen for the periventricular ischemic changes in both cases. Additional imaging included DaT imaging which showed bilateral asymmetric reduced nigro-striatal uptake in two cases with parkinsonism; this remained unchanged at ten months in the one who had a follow-up scan. Brain FDG PET in a myoclonus ataxia patient demonstrated putaminal and cerebellar hypermetabolism and diffuse cortical hypometabolism which normalised within eight weeks. Imaging findings from patients with persistent movement disorders are listed in the table. In summary, imaging did not reveal significant differences between the two groups that could explain the outcome. Data on CSF findings and neurophysiologic results were incomplete so they could not be used for correlation anaylsis.

## Discussion

Movement disorders as a manifestation of COVID-19 appear to be rare; with myoclonus ataxia syndromes being the most common. However, parkinsonism, dystonia, chorea and other movement disorders have also been reported. To date, case reports largely lack follow-up data, yielding uncertainty about their long-term outcome. Here we provide follow-up information of 27 patients who had developed a new-onset organic movement disorder in the context of their COVID-19 infection. We found that the prognosis of the movement disorder is generally good, with the majority recovering without residual symptoms within several weeks or months. It remained unclear why ataxia and myoclonus recovered earlier than parkinsonism and chorea. Only in a fraction of patients (6 of 27; 22%) the movement disorder was persistent after a follow-up period of 7.5 ± 3 weeks. A severe clinical course of COVID-19 in general and the development of an encephalopathy were risk factors for the persistence of the movement disorder, albeit others who had a severe course recovered so this is not a strong predictor. Given the small number of patients, data on the presence or absence of comorbidities should be interpreted carefully.

Brain imaging studies were done in a heterogenous pattern (i.e. MRI vs CT, different sequences, at different times, with or without follow-up etc.)—which owes to the different clinical practices (and availability of imaging machines) across countries. It appears that results were unremarkable in two thirds of patients. Reported abnormalities included cortical, cerebellar and brainstem ischemic and meningeal enhancement. Follow-up information was scarce, available only for eight patients, in some of whom imaging abnormalities had resolved or remained static. DaT imaging was done in two case and revealed reduced uptake which remained unchanged after 10 months in the one where assessed.

Previous literature suggested that myoclonus may develop fairly soon after the initial infection, whereas parkinsonism manifests later [4, 18]. Our data do not recapitulate this observation. Thus, clinicians should be alert for the different types of movement disorders early and late after the initial infection.

Different hypotheses regarding the pathogenesis for acute COVID-19-associated movement disorders have been formulated, including anatomical, i.e. strategic, lesions (e.g. due to hypoxia), drug-induced, cytokine-mediated or as an autoimmune process. Accordingly, the prognosis may differ between cases. Thus, drug-induced cases may be more likely to recover once the triggering agent is removed [17], compared to someone with a secondary movement disorder associated with a hypoxic brain lesion.

About half of the patients were treated with some form of immunomodulatory treatment. In our series patients with hyperkinetic movement disorders received immunomodulation, while those with parkinsonism or isolated ataxia or those with suspected hypoxic ischemic encephalopathy did not receive immunomodulation.The decision whether to introduce this type of treatment was made by the treating physician based on clinical experience and local operating procedures. We are not aware of autoantibody positivity which may have influenced this decision; and based on the uncontrolled nature of our data conclusions in how far immunomodulatory treatment influenced time of recovery or prognosis cannot be drawn.

It is encouraging that almost three years into the pandemic only a handful of patients with persistent movement disorders have been reported. The concern is raised in view of the outbreak of encephalitis lethargica and postencephalitic parkinsonism in the 1920s which occurred after the 1918 influenza pandemic, commonly referred to as the Spanish flu. A second-hit mechanism has been proposed, however, the causal link remains a matter of debate, particularly with a lack of comprehensive surveillance data. For COVID-19, great vigilance is recommended in order to timely recognize and address potential neurological long-term manifestations of SARS-CoV2, including parkinsonism, especially in the long-term. Post-COVID movement disorders reflect a heterogeneity of mechanisms and thus one’s ability to draw firm conclusions on the (long-term) outome is limited.

The major limitation of our study is the relatively low response rate of 33%. Reasons for this could be the unavailability of follow-up as the patients may have died without informing the corresponding author, may have been admitted under other departments or a limited course of their disease. Notably, this leads to potential interpretation bias.

Finally, the cases reviewed here are all thought to be organic by nature. Notably, functional movement disorders associated with the SARS-CoV2 pandemic, lockdown and vaccine-related stressors have also been observed and reported recently. These include rapid-onset functional tic-like behaviours. The prognosis of the latter is good in affected adolescents, but less so in affected adults, based on a recent prospective follow-up study over 6 months [19].

Thus, our global multicentre collaborative study assessing long term outcome of post-COVID movement disorders suggests that the majority of the hyperkinetic movement disorders and immune mediated ataxias are self-limiting, while parkinsonism or ataxia due to hypoxia, ischemia or severe demyelination are more likely to take a chronic static persistent course requiring long-term management.

## Supplementary Information

Below is the link to the electronic supplementary material.

Video 1: This 62-year old patient’s COVID-19 infection manifested with fever, headache, nausea, and diarrhea in July, 2021. His symptoms resolved within 10 days except for “shaking” in both hands. On admission he was confused, hallucinating and displayed generalized stereotyped movements of his whole body. EEG and  brain MRI were normal. He gradually spontaneously improved but was left with residual tremor affecting both his upper and lower extremities while at rest and during action (shown in the video) but no evidence of bradykinesia (MP4 8004 KB)

Video 2: This 71-year old male patient presented due to confusion and gait disturbance. On admission he had generalized action-induced myoclonus affecting his limbs and face (see video) which improved over the course of 14 days (MP4 45776 KB)

## Data Availability

The datasets generated during and/or analysed during the current study are available from the corresponding author on reasonable request.
